# Multiscale imaging of plants: current approaches and challenges

**DOI:** 10.1186/s13007-015-0050-1

**Published:** 2015-02-10

**Authors:** David Rousseau, Yann Chéné, Etienne Belin, Georges Semaan, Ghassen Trigui, Karima Boudehri, Florence Franconi, François Chapeau-Blondeau

**Affiliations:** Université de Lyon, Laboratoire CREATIS, CNRS, UMR5220, INSERM, U1044, Université Lyon 1, INSA-Lyon, Villeurbanne France; Laboratoire Angevin de Recherche en Ingénierie des Systèmes (LARIS), Université d’Angers, 62 avenue Notre Dame du Lac, Angers, 49000 France; GEVES, Station Nationale d’Essais de Semences (SNES), rue Georges Morel, Beaucouzé, 49071 France; La Plateforme d’Ingénierie et Analyses Moléculaires (PIAM), Université d’Angers, Angers, 49000 France

**Keywords:** Mutiscale imaging, Multiscale filtering, Wavelets, Fractal, ImageJ plugins

## Abstract

We review a set of recent multiscale imaging techniques, producing high-resolution images of interest for plant sciences. These techniques are promising because they match the multiscale structure of plants. However, the use of such high-resolution images is challenging in the perspective of their application to high-throughput phenotyping on large populations of plants, because of the memory cost for their data storage and the computational cost for their processing to extract information. We discuss how this renews the interest for multiscale image processing tools such as wavelets, fractals and recent variants to analyse such high-resolution images.

## Introduction

Finding the good practices to perform high-throughput phenotyping of large populations of plants is a current challenge to meet the high-throughput capacity of genotyping and push forward the knowledge on the development of plants in different environments. Because they allow contactless and noninvasive measurements, imaging techniques are regarded as tools of highest interest in this context, to provide anatomical or physiological objective traits and outperform the limit of human vision either in terms of sensitivity, accuracy or throughput. Conversely, plant sciences constitute a new field of application for computer vision which traditionally, when applied in life sciences, used to focus more on biomedical imaging. Among the specificities of computer vision for plant sciences that are not found in biomedical imaging, is the possibility to monitor, continuously over the whole life cycle, the process of growth on structures possessing complex 3D multiscale organisation with a part visible in the air (shoot) and a part hidden in the soil (root).

There has been a significant increase in interest in plant imaging and image analysis methods in recent years, but most of the techniques proposed focus on measurements at a single scale - cell, organ, whole plant, etc. This is in contrast to modelling efforts which have stressed multiscale approaches. Such numerical models have been proposed at the scale of the entire structure of plants from iterated replication processes using L-systems (see [1,2] for reviews). Such replication processes have been shown able to reproduce the fractal organization of plant structures as measured on entire real plants. These can also serve to model the root systems [3] and have recently been used to validate image processing algorithms for root segmentation [4]. Multiple plant modeling coupled to agronomical models have also been developed [5] and allow the numerical validation of image processing algorithms at the scale of canopy. Replication processes have also been modeled at the cellular scale with possibilities of explanatory physical mechanisms for the shape of the plant at higher scales [6]. As another instance, the so-called dead leaves model takes inspiration from the foliage of plants, with leaves of different sizes and illumination which are reproduced at various scales with occlusions [7-9]. Such models have been shown to produce fractal patterns with controllable properties, and in return they offer models for the multiscale constitution of plants.

Due to the increase in size and resolution of the imaging sensors and to the development of efficient registration methods, the number of scales accessible in imaging is now ready to meet the multiscale structure of plants. In this review article, we present a set of recent high-resolution imaging techniques which cover the plant scales from molecules in the cell up to the field, and we detail how this renews the interest of scale-analysis tools for image processing.

## Multiscale high-resolution imaging in plant sciences

**Figure 1:**
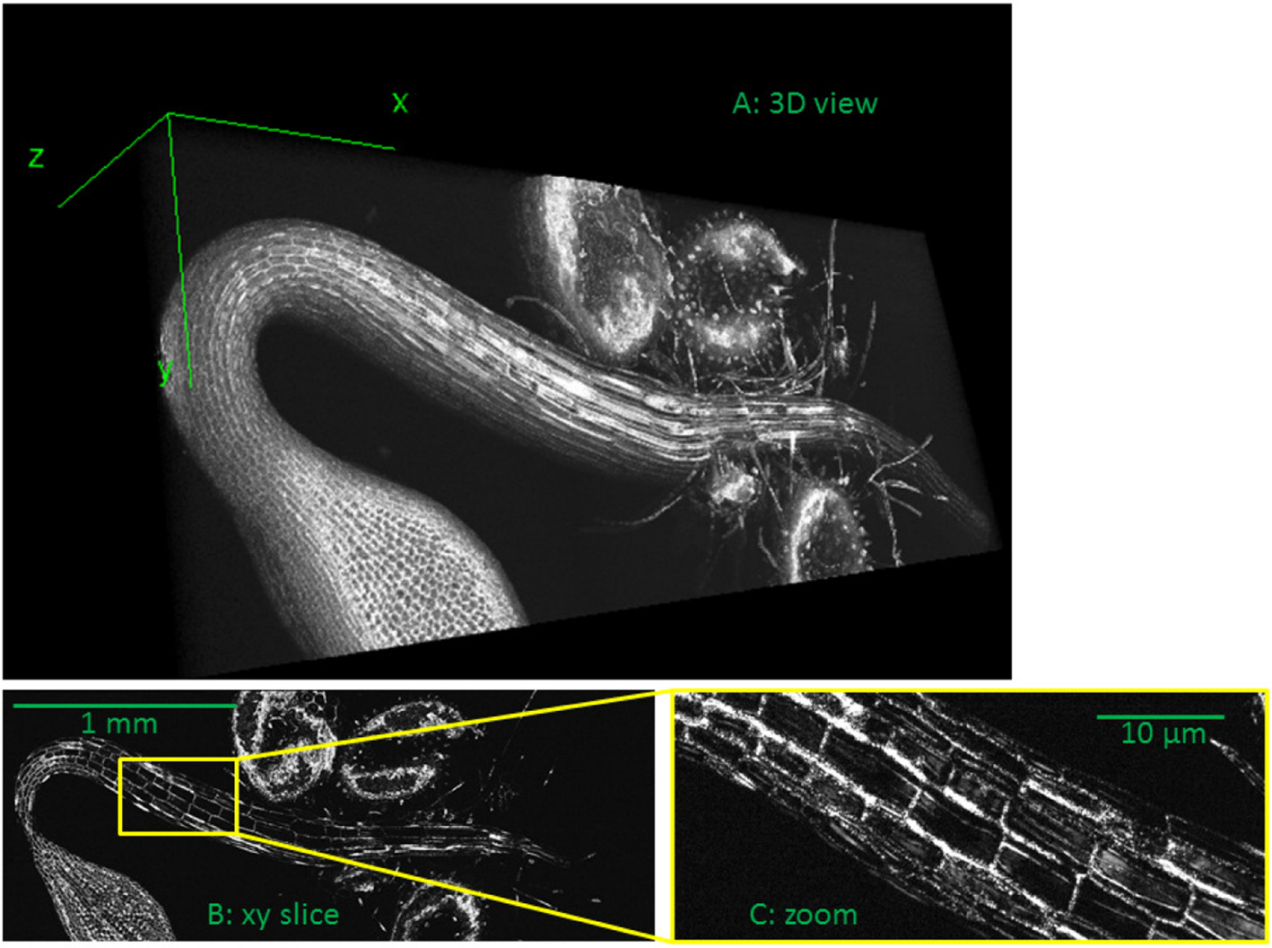
**Images of a seedling of** ***Arabidopsis thaliana*** **acquired with optical coherence tomography (see [** 31 **] for another illustration of OCT with plants).** Panel **A**: 3D view of an entire seedling. Panel **B**: 2D view in XY. Panel **C**: zoom in the solid rectangle of the 2D view of panel **B**.

We give in Table 1 a list of imaging techniques which have been shown in the recent literature to cover multiple scales of interest for plant sciences. At the smallest scales, single molecules up to the the entire cell are now also accessible for plants [10] with super-resolution imaging techniques [11] outperforming the classical diffraction limits such as photoactivated localization microscopy (PALM), stochastic optical resolution microscopy (STORM) [12], stimulated-emission depletion microscopy (STED) [13], three-dimensional structured illumation microscopy (3D-SIM) [14] and total internal reflection fluorescence microscopy (TIRF) [15]. At a higher range of scales, some recent microscopic imaging techniques now allow to discriminate cells of an entire organ. This is illustrated in Figure 1 with an example of optical coherence tomography (OCT) of a seedling of *Arabidopsis thaliana* during elongation with a resolution enabling to discriminate the cells of the seedling and the entire seedling. Other microscopic imaging techniques also have this capability and have been applied to plants like X-ray phase contrast imaging (X-ray PCT) [16] for microstructure analysis of the voids in an entire seed, light sheet fluorescence microscopy (LSFM) [17], multiangles confocal microscopy [18] to observe the entire seedling growth cell by cell, or optical projection tomography (OPT) [19] to image an entire leaf with possibility of cell resolution. At larger scales, inside the soil, imaging techniques give access to nodules on the root system up to the entire root system. This has been recently demonstrated in 3D in soil with absorption-based micro X-ray computed tomography [20-22], and with high-resolution imaging in 2D with rhizotron using reflectance imaging [23], or with bioluminescence imaging [24,25]. At the same metric scales but in the air, imaging techniques give access to a leaf in the shoot up to the entire shoot. This has been recently demonstrated with a variety of 3D imaging systems (see [26,27] for a recent reviews). At still larger metric scales, in field conditions, one can capture with high-resolution imaging setups embedded on an airplane or unmanned aerial vehicle (UAV) [28,29] the entire shoot from top view up to the canopy constituted by assemblies of shoots.

**Table 1:** **Multiple scale high-resolution imaging in plant sciences**

**Biological scales**	**Metric scales**	**Imaging techniques**
From molecule to cell	10 nm to 10 *μ*m	PALM-STORM [12],
		STED [13], 3DSIM [14]
From cell to organs	0.1 *μ*m to dcm	OCT [30], LSFM [17],
		X-ray PCT [16],
		confocal [18], OPT [19]
From nodules to root system	*μ*m to m	Rhizotron [24,25],
		X-ray *μ*CT [20-22]
From leaf to entire shoot	mm to 10 m	depth-imaging,
		LIDAR [26,27]
From shoot to canopy	m to hm	remote sensing,
		UAV imaging [28,29]

Acronyms are explicated in Section “Multiscale high-resolution imaging in plant sciences”.

The list of imaging techniques given in Table 1 is not exhaustive (see [32-34] for recent reviews). This familly of new imaging systems bring some challenges that would be interesting to be discussed in the field of instrumentation when applied to plants. To point only one, the new microscopies of Table 1 have been introduced for applications of broad interest in life sciences and often demonstrated on organisms which serve as models for biology, such as C-elegans, zebra fish, mice, *Drosophilae* fly or *Arabidopsis thaliana*... Consequently, the non-invasiveness property of the light used to acquire images of such a variety of organisms is mainly expressed as nonphototoxic if it does not kill the organism on a time scale linked with the time required by instrumentation for image acquisition. For specific applications on plants however, imaging the development can necessitate long time-lapsed acquisitions. For instance, imbibition and germination of a seed take hours while elongation of a seedling several days. At these stages of development illustrated in Figure 1, plants are supposed to grow in dark conditions in the soil with no light exposure, as light strongly modifies the physiology of seedlings since it activates the process of photosynthesis. It would therefore be important to revisit, as recently done for seedling in [35], the concept of phototoxicity, by adapting wavelength, energy and duration of the light used by the family of mutiscale microscopies when applied to plants.

In this review, we rather put the stress on current approaches and challenges brought by new imaging systems at the level of image processing. The point here is that techniques of Table 1 have in common, although working at very different metric and biological scales, to produce images requiring a huge capacity of data storage. This is due to the increasing size, resolution and dynamic of imaging sensors, but also to the coupling of imaging systems with motorized scanning systems. By this coupling, multiple views can be acquired and registered to produce high-resolution imaging. Multiview imaging is common practice in remote sensing. This is now extending to the scale of a single plant with rotating plates, or at the scale of the cells with microscope scanners. For instance, the OCT system used to produce Figure 1 is associated to microstage translation systems, in such a way that the imaging technique can, after registration, capture in 3D and at the cell resolution, hundreds of such entire seedlings in a single run, resulting in some Giga bytes of data. This is 10^6^ more than what has to be stored for one single plant imaged with a standard imaging resolution. Such large images can still be opened by a software like ImageJ but image processing, even some basic ones, can become very slow. This high memory cost, specially in the perspective of high-througput phenotyping for large population of plants, calls for adapted approaches. We propose a review of the most prominent of them in the following.

## Image processing tools for multiscale imaging

### Combining modalities with different scales

**Figure 2:**
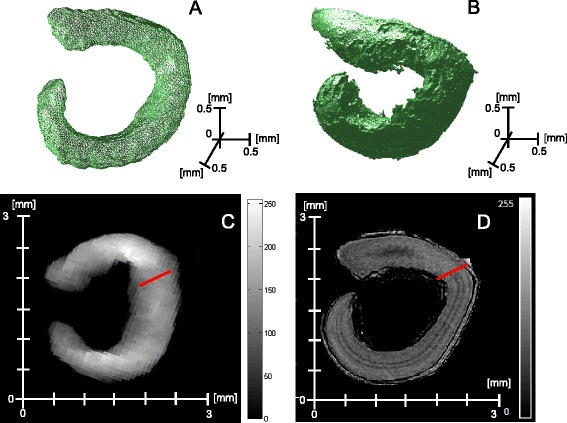
**Bimodal imaging of the embryo of a dry seed of sugar beet with a low spatial resolution of 0,187 mm per isotropic voxel in MRI (A external 3D view and C medial 2D slice) and high spatial resolution of 7,84** ***μ*** **m per isotropic voxel in X-ray tomography (B external 3D view and D medial 2D slice).** The MRI is a spin-echo sequence giving gray-level propotional to the lipid content of the embryo. The red line in panel **D** is positionned manually on the X-ray at the separation between cotyledon and radicle. Red line in panel **C** is automatically positioned after registration of both imaging modalities with the ImageJ plugin TrakEM2 of Table 2.

A problematic of current interest in multiscale imaging is to combine imaging modalities providing different scales and contrasts. This association has for instance been illustrated in plant sciences with electron microscopy combined with confocal microscopy [36], or magnetic resonance imaging (MRI) combined with positon emission tomography (PET) [37] or again depth imaging combined with thermal imaging [26]. In these examples one of the modality has a relative high spatial resolution (electron microscopy, MRI, depth imaging) which provides an anatomical information while the other modality (respectively confocal, PET, thermal imaging) gives a more functional information. The functional modality can be used to locate a region a interest to be further analysed from an anatomical point of view or the other way round. This is a useful way to reduce the amount of data to be explored at high resolution. Also, the high-resolution modality can be used to analyze separately different anatomical compartments, not clearly contrasted in the functional modality. This is illustrated in Figure 2 where a 3D image of sugar beet dry seed has been acquired with a high-resolution X-ray tomograph and a MRI sequence providing gray levels proportional to the content of lipid in the seed. This gives an image of the embryo of the dry seed. As shown in Figure 2, the high-resolution modality can be used to identify the position of the cotyledon and the radicle in the embryo. If the two modalities are registered, the landmark corresponding to the beginning of the separation between cotyledon and radicle can be applied onto the MRI images and then allowing a comparison of the lipid content of these two sub-organs of the seed. Specifically here, this shows the expected higher content of lipid in the cotyledon than in the radicle. The registration step is a key image processing step in this combination of modalities. Image registration is a problematic of image processing by itself [38] with various approaches (conventionnally classified as rigid versus non rigid, automatic versus manual,...) which have in common the calculation of a transformation matrix to be applied on one of the modality so as to be able to surperimpose both modalities with a locally accurate match all over the images. The development of high-resolution multiscale images has called for the design of approaches adapted to the computational cost due to the large size of the images to be registered. Instead of performing the computation of the registration on the whole image, the transformation matrix is computed on a region of interest containing landmarks and then applied on the entire image (this is available in the ImageJ Plugin TurboReg pointed in Table 2). These landmarks can be selected manually or detected automatically with scale invariant feature transforms (SIFT) [39] or variants implemented in the ImageJ Plugin TrakEM2 pointed in Table 2. Random local deformation can occur with electron microscopy due to slicing or with MRI due to the so-called blooming effect or also with thermal imaging due to the presence of mixed pixels on edges of structures. The compensation of these local deformations randomly occuring in one of the imaging modalities remains an open challenge for image registration.

**Table 2:** **Multiple scale image processing tools available under the free and open software ImageJ**

**Image processing task**	**ImageJ plugin weblink**
Image registration	http://fiji.sc/TrakEM2
Landmark detection	http://fiji.sc/Feature_Extraction
Wavelet filtering	http://bigwww.epfl.ch/demo/fractsplines/java.html
Multiscale blob extraction	http://bigwww.epfl.ch/sage/soft/Log3D/
Multiscale vessellness extraction	http://www.longair.net/edinburgh/imagej/tubeness/
Nonlocal mean denoising	https://code.google.com/p/ij-non-local-means/
Fractal analysis	http://rsb.info.nih.gov/ij/plugins/fraclac/
Multiscale color analysis	http://www.signal-image.net/2010/04/color-inspector-3d/

### Selecting scales

The selection of structures appearing in the images at given scales can be realized with filters. The design of these filters has to incorporate some prior knowledge on the shape of the objects to be found at each scale. Among the strategies for the design of bank of filters, wavelets have shown to be a very powerful approach for application in plant sciences, see [40] for a review of the late 90’s, which continue to be investigated to select patterns on leaves [41-44] or on canopies [45,46]. The wavelet approach is versatile since a large panel of wavelet functions have been designed. A wavelet is a wave-like oscillation with an amplitude that begins at zero, increases, and then decreases back to zero on a scale which can be defined by the user. Such functions are expected to constitute good filters when they share common features with the shape of the objects to be extracted. Some familly of filters have specifically been developed to extract given shapes. Let us shortly underline the vesselness filter [47] which enhances area in images where the gradient in the image is almost null in one direction and much higher in the other perpendicular directions. This situation is found with any tubular structures. Therefore, although initially developed to enhance biomedical images with vascular vessels the vesslness filter is also very much suited to enhance tubular structures met in plant sciences such as cell walls, leaf veins or branches in trees. Based on the same philosophy, enhancing areas with high gradient in all directions extract the blob-like structures [48] (cells, nodules, spherical fruits, …), or also enhancing areas with high gradient in only one direction in space extracts surface-like structures (cellular layers, plant leaves,…). These familly of filters are available under ImageJ as mentioned in Table 2.

When no prior knowledge on the shapes or scales of the objects of interest in the image is available, it is necessary to use self-adaptive methods to automatically select the appropriate scales of interest. Such methods are known as wavelet packets decomposition. However, in this case, the choice of the wavelet and of the range of scales to be analysed still have to be performed by the user. Another self-adaptive method, of more recent introduction, is the empirical mode decomposition also called Hilbert-Huang transform, where the scale analysis is purely based on the data itself. Data-dependent modes, corresponding to the local frequency data, are extracted by the analysis to decompose the signal, instead of a decomposition on preexisting elemental functions such as wavelets. Introduced for monodimensional signals [49], empirical mode decomposition has then been extended to images [50] and successfully applied to texture characterization [51]. The dominant modes of the decomposition single out the main scales in the signals or images under analysis, and keeping only the dominant modes offers natural methods for parsimonious representation and for data compression. Efficient compression schemes have been developed for landscapes captured in remote sensing for scales from canopy to field [52]. Such compression approaches by scale selection remain open for investigation for the other scales of Table 1.

**Figure 3:**
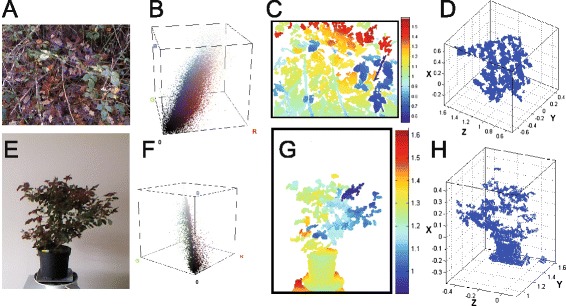
**Bimodal RGB-depth representation of a forestery scene (first row) and a single plant (second row).** Panels **A** and **E**: RGB luminance. Panels **B** and **F**: corresponding RGB histogram. Panels **C** and **G**: depth map expressed in meter. Panels **D** and **H**: corresponding point cloud of the depth map.

Another active field of image processing associated to the selection of scales is image denoising. Benchmark are found in the litterature [53] so as to identify the best techniques. However, such benchmarks are mostly organized on natural images not specifically suited for a given scientific field. It is very likely that the ranking of best practices may vary depending on the specific type of images. A specificity of multiscale images in plant sciences is the presence of replicated structures. This is visible in Figure 1 with cells or in Figure 3 with leaves. This replication process found in plant architecture constitutes a prior which is not found in all natural images. This observation motivates the choice of the so-called nonlocal mean [54] as interesting denoising methods. Nonlocal mean denoising is realized by averaging pixel content weighted by how similar these pixels are to the target pixel. In its principle, this non local averaging process, available under ImageJ plugin given in Table 2, will be very efficient if a lot of pixels are similar to the target pixel like in the self-similar structures found in plant sciences.

### Characterizing multiscale signatures

Instead of selecting specific scales of interest, another approach is to characterize the global organization over multiple scales in the images. Nontrivial regularities developing in a self-similar way across a significant range of scales usually identify the existence of a fractal organization. Fractal concepts have been shown relevant to the description of plants, of their roots and shoots, which often show self-similar organizations across scales [55-59]. Especially, such organizations lead to high surface areas at the interfaces with the environment, ensuring for the plant efficient capture of nutrients and energy.

Self-similarity accross scales, i.e. fractal features, can thus be found in various properties of images from plants. For instance, they have been reported in the spatial organization of gray-level luminance images from outdoor scenes of woods and plants [60,61]. This is manifested by scale-free power-law evolutions present in the frequency spectrum of luminance images or also in their spatial correlation functions. Also, the colorimetric organization of natural images including landscapes with plants has been reported to carry self-similarity and fractal properties [62-64]. More recently, multiscale analysis has been undertaken for plant images obtained from another imaging technique delivering depth images of a physical scene [65]. The depth map images from outdoor scenes of woods and plants as in [60,61] were shown in [65] to also reveal self-similarity and fractal properties. Such multiscale image analyses revealing and characterizing fractal properties in plants are important to contribute to their understanding, since the fractal and multiscale organization of plants has a direct impact on their functioning, for instance for efficient interactions with their environment as evoked above [58,66-68]. Also, fractal characterization of plants is useful to devise synthetic models of plants with sufficient realism [1].

**Figure 4:**
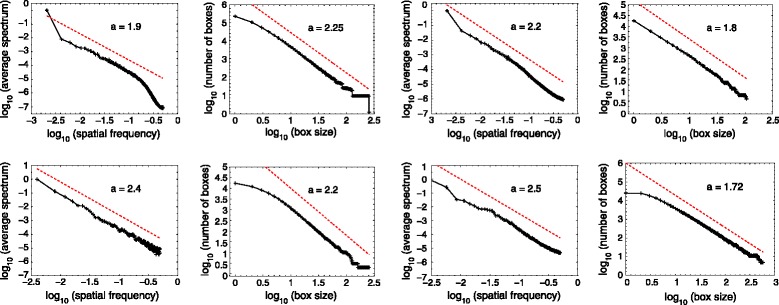
**First and second rows: multiscale analysis of RGB-depth images of first and second rows of Figure** 3 **.** First column: average spectrum of RGB luminance image as a function of spatial frequency on a log-log plot. Second column: box counting in the RGB histogram as a function of the box size on a log-log plot. Third column: average spectrum of depth map image as a function of spatial frequency on a log-log plot. Fourth column: box counting in the point cloud of the depth map as a function of the box size on a log-log plot. In each graph, the dotted line with its slope indicated represents a model to appreciate a power-law evolution to match the data. The slopes reveal noninteger exponents for the power-law evolutions matching the data over a significant range of scales. This indicates nontrivial self-invariance of the data across scales, i.e. a fractal organization.

For illustration, we proceed to the scale analysis of several images from a forestry scene and from a single plant, as shown in Figure 3, acquired with a bimodal RGB-depth camera [69]. Figure 3 shows four possible ways of vizualizing such data, with an RGB luminance image, with a 3D RGB histogram, with depth map or with a 3D depth point cloud. We analyze the scale organization in each of these four representations. The spatial frequencies of the RGB luminance images in Figure 3 are analyzed with the power spectrum computed via the periodogram method, through the squared modulus of the two-dimensional Fourier transform, expressed in polar coordinates in the plane of spatial frequencies from a single plant, as shown in Figure 3, acquired with a bimodal RGB-depth camera [65]. An average is then realized over the angular coordinate to yield the orientationally averaged spectrum. This power spectrum is computed on a gray-level version of the RGB image of Figure 3 and on the depth image of Figure 3 as a function of the spatial frequency. The results shown in Figure 4 demonstrate for both the forestry scene and for the single plant, and with both imaging modalities, scale-invariant power-law signatures over a significant range of scales, represented by the spatial frequency. Also, in Figure 4 we implemented the box counting method [65] on the point clouds constituted by the RGB histogram of Figure 3 and by the depth image of Figure 3. The box counting values are obtained in terms of scales represented by the side length of the various boxes. For each side length, we compute the number of boxes with this side length which are needed to cover all the point cloud. Here again the results shown in Figure 4 demonstrate, for both scenes and both modalities, power-law signatures over a significant range of scales, represented by the size of the covering boxes. As shown in Figure 4, the measures computed in luminance space, in RGB space as well as in depth space, all display scale-invariant power-law signatures over significant ranges of scales. Such fractal signatures are interesting in the context of multiscale imaging since they constitute an efficient and concise way to characterize a complex organization. Fractal image processing tools have been widely applied to characterize plants (see [70] for a review) at the scales of leaf [67,71], canopy [72]. So far the fractal characterization of plants at the microscopic scale is open for investigation. Fractal analyses of root systems have been undertaken but mainly from plants taken out of the soil [73-75]. The new high-resolution X-ray CT reported in [20-22] therefore opens new perspectives for the fractal characterization of the root system directly in 3D and in the soil.

## Conclusion

High-resolution multiscale imaging in plant sciences was until recently limited to the domain of remote sensing. It is now also possible to capture entire roots or shoots of plants, at various stages of development, with cellular or subcellular spatial resolution. These high-resolution imagings are producing huge amounts of data, specially when they are applied to large populations of plants in high-throughput phenotyping. In this framework, we have highlighted here some current approaches connected to the multiscale analysis of plants and pointed toward efficient computational implementation under the free and open software ImageJ in Table 2. Open problems emerge for image compression and image characterization. Multiscale approaches are specifically relevant for the new microscopies such as those presented in Table 1; these are more recent and have received so far, in a multiscale perspective, less attention than remote-sensing imaging or than proximal detection in the field.
